# The Impact of Korean Medicine Treatment on the Incidence of Parkinson’s Disease in Patients with Inflammatory Bowel Disease: A Nationwide Population-Based Cohort Study in South Korea

**DOI:** 10.3390/jcm9082422

**Published:** 2020-07-28

**Authors:** Hyeonseok Noh, Jeongju Jang, Seungwon Kwon, Seung-Yeon Cho, Woo-Sang Jung, Sang-Kwan Moon, Jung-Mi Park, Chang-Nam Ko, Ho Kim, Seong-Uk Park

**Affiliations:** 1 Department of Clinical Korean Medicine, Graduate School, Kyung Hee University, 26, Kyungheedae-ro, Dongdaemun-gu, Seoul 02447, Korea; integritynoh@hanmail.net (H.N.); kkokkottung@hanmail.net (S.K.); sy.cho@khu.ac.kr (S.-Y.C.); wsjung@khu.ac.kr (W.-S.J.); skmoon@khu.ac.kr (S.-K.M.); pajama@khu.ac.kr (J.-M.P.); kcn202@khu.ac.kr (C.-N.K.); 2 Graduate School of Public Health, Seoul National University, 1, Gwanak-ro, Gwanak-gu, Seoul 151-742, Korea; zzooo@snu.ac.kr; 3 Department of Cardiology and Neurology, College of Korean Medicine, Kyung Hee University, 26, Kyungheedae-ro, Dongdaemun-gu, Seoul 02447, Korea; 4 Department of Public Health Science, Graduate School of Public Health & Institute of Health and Environment, Seoul National University, 1, Gwanak-ro, Gwanak-gu, Seoul 08826, Korea

**Keywords:** Parkinson’s disease, inflammatory bowel disease, Korean medicine, National Health Insurance Service-Senior cohort, nationwide population-based study

## Abstract

We aimed to investigate the association between Korean medicine (KM) treatment and the risk of Parkinson’s Disease (PD) in patients with inflammatory bowel disease (IBD) in South Korea. This study analyzed data from the National Health Insurance Service-Senior cohort in South Korea. The 1816 IBD patients enrolled in the analysis comprised 411 who received only conventional treatment (monotherapy group) and 1405 who received both conventional and KM treatments (integrative therapy group). The risk of PD in patients with IBD was significantly lower in the integrative therapy group than in the monotherapy group after adjusting for confounding variables (adjusted hazard ratio (HR), 0.56; 95% confidence interval (CI) = 0.34–0.92). In the mild Charlson Comorbidity Index (CCI) group, the risk of PD in patients with IBD in the integrative therapy group was 0.39 times lower (adjusted HR, 95% CI = 0.20–0.77) than that in the monotherapy group. However, there was no significant difference in the risk of PD in patients with IBD between the integrative therapy and monotherapy groups among individuals with severe CCI (adjusted HR, 0.90; 95% CI = 0.41−1.96). IBD patients are at a decreased risk of PD when they receive integrative therapy. KM treatment may prevent PD in IBD patients.

## 1. Introduction

Parkinson’s disease (PD), the most common movement disorder, is neuropathologically characterized by progressive loss of dopaminergic neurons and the presence of α-synuclein-containing Lewy bodies in the substantia nigra [1]. An estimated 1% of the population older than 60 years of age is affected by PD. The disease typically causes motor symptoms such as rigidity, resting tremor, bradykinesia, and postural instability, as well as non-motor features including constipation, depression, sleep disorders, and autonomic dysfunction. Interactions between environmental and genetic factors, including traumatic brain injury, pesticides, oxidative stress, and genetic mutations contribute to the risk of PD [2].

Inflammatory bowel disease (IBD), comprising Crohn’s disease (CD) and ulcerative colitis (UC), is a chronic and recurrent inflammatory disorder. Environmental and microbial factors may interact with genetic components in the pathogenesis of IBD [3]. Communication between the enteric and central nervous system (CNS) activity through the gut–brain axis is associated with immune activity modulation. Chronic intestinal inflammation may contribute to dopaminergic neurodegeneration by disruption of the blood–brain barrier (BBB) [4]. Gastrointestinal (GI) inflammation can trigger α-synuclein expression and accumulation in the gut, the aggregates of which subsequently propagate along the vagus nerve to the CNS. The dopaminergic neurodegeneration by 6-hydroxydopamine (6-OHDA) in the CNS may also induce GI tract inflammation by impairing the dorsal motor nucleus of the vagus nerve [5,6]. Several cytokines, including interleukin (IL)-1β and tumor necrosis factor (TNF)-α, have been associated with PD and IBD [7]. PD and IBD also share key genetic factors including caspase recruitment domain-containing protein 15/nucleotide-binding oligomerization domain-containing protein (*CARD15/NOD2*) gene polymorphisms as well as *LRRK2* mutations [6,7].

Several population-based studies examining the clinical co-occurrence of PD and IBD have reported an increased risk of PD in patients with IBD [8,9,10,11,12]. However, previous studies had limitations and conflicting results regarding the incidence of PD due to IBD in terms of medication use, comorbidities, and personal health behaviors. To our knowledge, no studies have compared the incidence of PD in IBD patients who received healthcare services other than conventional treatment. Besides conventional medication therapy, Korean medicine (KM) treatment, such as acupuncture, moxibustion, cupping, psychotherapy, and insured herbal preparations has been widely used for the treatment of IBD in South Korea. Therefore, this nationwide population-based study aimed to investigate the association between KM treatment and the risk of PD in patients with IBD in South Korea and provide evidence for the preventive advantage of KM treatment on the incidence of PD in patients with IBD.

## 2. Methods

### 2.1. Data Sources

This retrospective population-based cohort study was based on data from the nationwide administrative claims-based database of the National Health Insurance Service (NHIS) in South Korea. The NHIS database is categorized into insured employees, insured self-employed individuals, or medical aid beneficiaries and includes information on personal demographics, inpatient and outpatient medical services, prescription records, and procedures and diagnosis according to International Classification of Diseases, 10th revision (ICD-10) codes [13]. Health-related information obtained using questionnaires (physical activity, alcohol consumption, cigarette smoking, coffee drinking, and self-reporting of past medical history and family history), anthropometry, blood pressure measurements, and clinical laboratory results were also included in the general health screening database. Since the data were de-identified, subject consent was not required.

The NHIS-Senior cohort statistically represents the population aged more than 60 years selected by 10% simple random sampling from a total of approximately 5.5 million South Korean subjects aged 60 years and over in 2002. The cohort was followed until 2015 unless death or disqualification for National Health Insurance, such as emigration [14]. The study protocol was exempted from the Institutional Review Board (IRB) of Kyung Hee University Korean Medicine Hospital at Gangdong (IRB No. 2019-09-009).

### 2.2. Study Population

Patients diagnosed with IBD were identified based on ICD-10 codes (K50 for CD and K51 for UC) as the primary or secondary diagnosis between 1 January 2002, and 31 December 2006, from the NHIS-Senior cohort database according to two or more outpatient clinic visits or hospitalization for more than two days. All IBD patients were followed up from 1 January 2007, to 31 December 2015. Patients with PD following IBD diagnosis were defined according to ICD-10 codes G20 during the follow-up period. The exclusion criteria were primary or secondary diagnoses of PD prior to IBD or death from 1 January 2002, to 31 December 2006.

### 2.3. Definitions of Variables

The baseline characteristics investigated in this study included sex, age, comorbidities, Charlson Comorbidity Index (CCI), medication use, hospitalization (days), outpatient use (days), and number of hospitals visited. As described previously, the comorbidities considered in this study included alcohol-related diseases (ICD-10 codes F10 and K70), cardiovascular diseases (ICD-10 codes I21–22, I252, I099, I110, I130, I132, I255, I420, I425–I429, I43, I50, G45–46, I60–I69, H340), chronic kidney diseases (ICD-10 codes N032–N037, N052–N057, N18–19, N250, I120, I131, Z490–Z492, Z940, Z992), chronic obstructive pulmonary disease (COPD) (ICD-10 codes J40–J49), dementia (ICD-10 codes F00–F03, G30, G31), depression (ICD-10 codes F32–33, F34.1), diabetes mellitus (ICD-10 codes E10–E14), hyperlipidemia (ICD-10 codes E780–E785), and hypertension (ICD-10 codes I10–I15) [15,16,17,18,19]. According to the CCI, the severity of the comorbidities was stratified as mild (0 or 1 point) or severe (2 points or more). Information about the drugs used to treat IBD (5-aminosalicylic acid, corticosteroid, immunomodulator, anti-TNF-α agent) is described in Supplementary Table S1 [20,21].

### 2.4. Exposures and Outcomes

The primary exposure examined was the cumulative use of KM treatment during the follow-up period. Each IBD patient’s exposure to KM treatment was determined using the National Health Insurance Electronic Data Interchange (EDI) codes provided by the Health Insurance Review & Assessment Service. The types of KM were classified as acupuncture, moxibustion, cupping, psychotherapy, and insured herbal preparations (Supplementary Table S2). The eligible IBD patients were divided into a monotherapy group that received only conventional treatment and an integrative therapy group that received both conventional and KM treatments. The outcome was newly developed PD (ICD-10 codes G20) as a primary or secondary diagnosis between 1 January 2007, and 31 December 2015, or until withdrawal from the insurance system.

### 2.5. Statistical Analysis

The differences in baseline characteristics between the two groups were compared by the t- and chi-square tests for continuous and categorical variables, respectively. The incidence rates of PD per 1000 person-years were calculated in each group. Cox proportional hazard regression analysis was used to calculate the adjusted hazard ratio (HR) and 95% confidence interval (CI) to determine whether KM treatment was an independent risk factor for the incidence of PD in patients with IBD after adjusting for sex, age, comorbidities, CCI, medication use, hospitalization (days), outpatient use (days), and number of hospitals visited. The cumulative incidences of PD between two groups were analyzed using the Kaplan–Meier method, and log-rank tests were used to compare differences in the curves. *p*-values < 0.05 were considered statistically significant. Statistical analysis was performed using SAS for Windows (version 9.4, SAS Institute, Inc., Cary, NC, USA) and R version 3.5.2 for Windows (R Core Team, Vienna, Austria, 2018).

## 3. Results

### 3.1. Study Description

**Figure 1:**
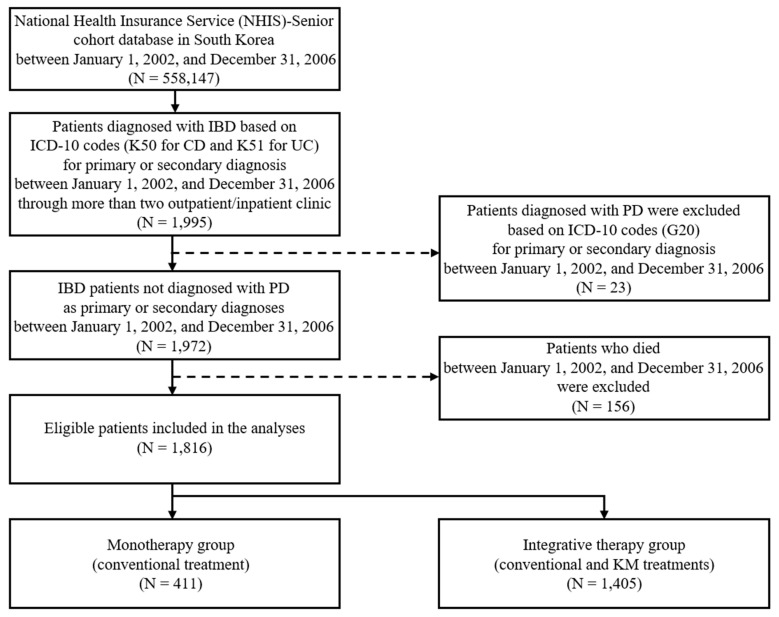
Flowchart of the study population. CD: Crohn’s disease, IBD: Inflammatory bowel disease, ICD-10: International Classification of Diseases, 10^th^ revision, KM: Korean medicine, PD: Parkinson’s disease, UC: Ulcerative colitis.

A total of 558,147 individuals over 60 years of age were identified in the NHIS-Senior cohort database from 1 January 2002, to 31 December 2006. Among them, 1995 were diagnosed with IBD based on ICD-10 codes (K50 for CD and K51 for UC) as primary or secondary diagnosis during the study period. After excluding 23 patients with pre-existing PD and 156 patients who died during the baseline period, the remaining 1816 eligible patients included 411 who received only conventional treatment (monotherapy group) and 1405 who received both conventional and KM treatments (integrative therapy group). The inclusion and exclusion criteria of the study population are shown in Figure 1.

### 3.2. Baseline Characteristics of the Study Population

The demographics of the study population at baseline are presented in Table 1. There were significant differences between the two groups in sex, COPD, CCI, and medication use (*p*-value < 0.001). The distributions of the other variables did not differ significantly between the two groups.

### 3.3. Incidence and Risk of PD in Patients with IBD According to KM Treatment

**Figure 2:**
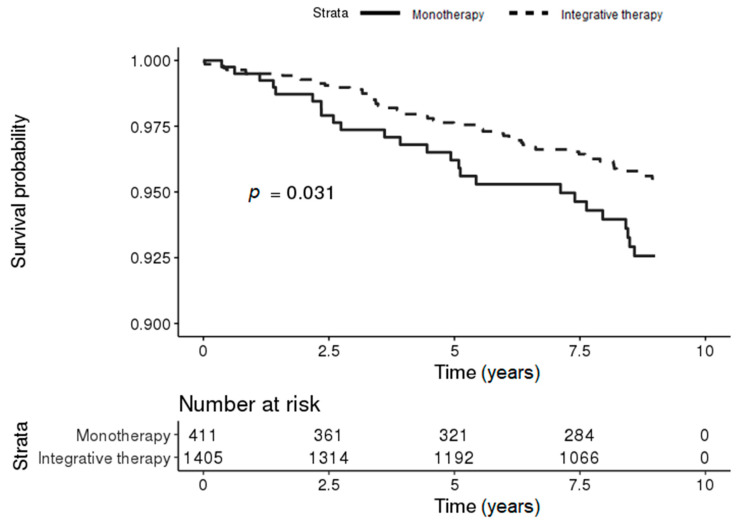
Kaplan–Meier survival curves for the incidence of PD in patients with IBD according to treatment (conventional [monotherapy] vs. integrated [conventional and Korean medicine]).

During the follow-up period, 55 (3.91%) and 25 (6.08%) patients in the integrative therapy and monotherapy groups, respectively, developed PD. The Kaplan–Meier survival curves for the risk of PD in patients with IBD differed significantly between the two groups, and the risk of PD in patients with IBD was significantly lower in the integrative therapy group (log-rank *p*-value = 0.031) (Figure 2).

IBD patients aged 65 years and over in the integrative therapy group were 0.51 times less likely to develop PD compared to the risk in the monotherapy group after adjusting for sex, comorbidities, medication use, hospitalization (days), outpatient use (days), and number of hospitals visited (95% CI = 0.28–0.94; 6.5 vs. 12.5 per 1000 person-years). Moreover, IBD patients in the integrative therapy group who used medication had a 0.54-fold lower risk of PD than those in the monotherapy group after adjusting for sex, age, comorbidities, hospitalization (days), outpatient use (days), and number of hospitals visited (95% CI = 0.30–0.96; 4.7 vs. 8.3 per 1000 person-years). After adjusting for confounding variables, the adjusted HR for developing PD in patients with IBD was 0.56 times lower in the integrative therapy group than in the monotherapy group (95% CI = 0.34–0.92; 5.1 vs. 8.4 per 1000 person-years) (Table 2).

### 3.4. Sensitivity Analysis

The sensitivity analysis showed that IBD patients in the integrated therapy group treated with KM more than twice had a 0.51-fold lower risk of PD than patients in the monotherapy group after adjusting for confounding variables (95% CI = 0.31–0.82; 4.9 vs. 8.8 per 1000 person-years). Cox regression analysis also showed that the risks of PD occurrence in IBD patients in the integrated therapy group who received KM more than three and more than five times were 0.57-fold (95% CI = 0.35−0.92; 5 vs. 8 per 1000 person-years) and 0.62-fold (95% CI = 0.39–0.98; 5 vs. 7.5 per 1000 person-years) lower, respectively, than those in the monotherapy group after adjusting for confounding variables. No statistically significant difference in the risk of PD in patients with IBD was observed between the integrative therapy and monotherapy groups among IBD patients treated with KM more than 10 times (adjusted HR, 0.68; 95% CI = 0.43−1.07; 5 vs. 6.7 per 1000 person-years) (Table 3).

### 3.5. Subgroup Analyses

The subgroup analyses divided the eligible IBD patients into two groups according to comorbidity severity based on the CCI (mild: 0 or 1 points, severe: 2 points or more).

#### 3.5.1. Comparison of the Incidence of PD in Patients with IBD According to KM Treatment in the Mild CCI Group

**Figure 3:**
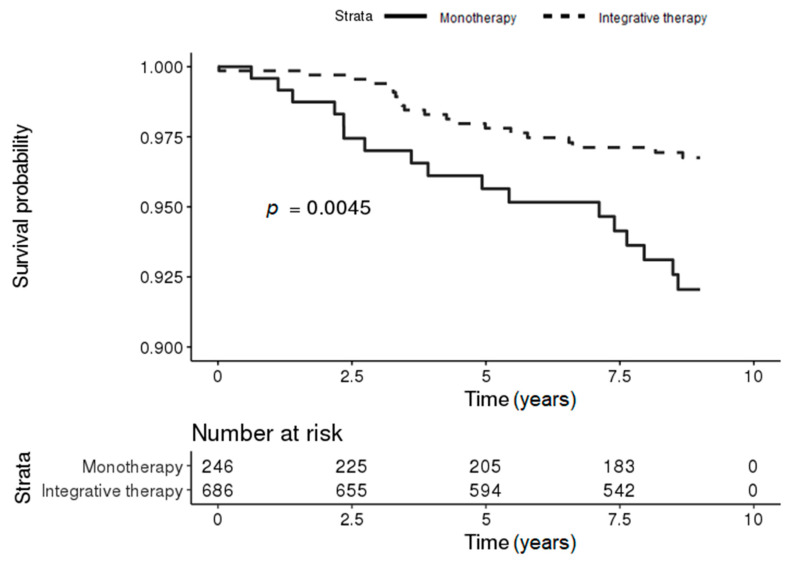
Kaplan–Meier survival curves for the incidence of PD in patients with IBD according to KM treatment in the mild Charlson Comorbidity Index (CCI) group (conventional [monotherapy] vs. integrated [conventional and Korean medicine]).

In the mild CCI group, 20 (2.92%) and 17 (6.91%) IBD patients in the integrative therapy and monotherapy groups, respectively, developed PD during the follow-up period. The Kaplan–Meier survival curves for the risk of PD in patients with IBD differed significantly between the two groups, and the risk of PD in patients with IBD was significantly lower in the integrative therapy group (log-rank *p*-value = 0.0045) (Figure 3).

#### 3.5.2. Comparison of the Incidence of PD in Patients with IBD According to KM Treatment in the Severe CCI Group

**Figure 4:**
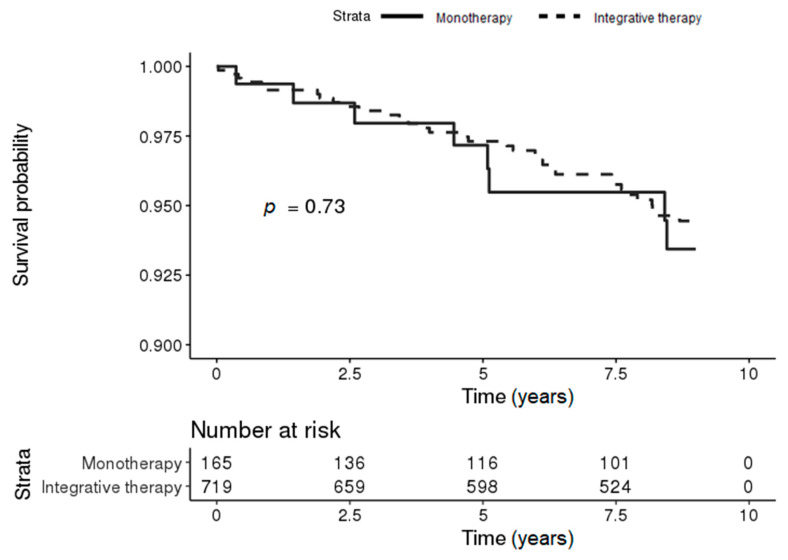
Kaplan–Meier survival curves for the incidence of PD in patients with IBD according to KM treatment in the severe CCI group (conventional [monotherapy] vs. integrated [conventional and Korean medicine]).

In the severe CCI group, 35 (4.87%) and 8 (4.85%) IBD patients the integrative therapy and monotherapy groups, respectively, developed PD during the follow-up period. The Kaplan–Meier survival curves for the risk of PD in patients with IBD showed no significant difference between the two groups (log-rank *p*-value = 0.73) (Figure 4).

#### 3.5.3. Risk of PD in Patients with IBD According to KM Treatment in the CCI Group

In the mild CCI group, Cox regression analysis showed that the risk of PD in patients with IBD in the integrative therapy group was 0.39 times lower than that in the monotherapy group after adjusting for confounding variables (95% CI = 0.20–0.77; 3.7 vs. 9.1 per 1000 person-years). After adjusting for confounding variables, no statistically significant difference in the risk of PD in patients with IBD was observed between the integrative therapy and monotherapy groups among individuals with severe CCI (adjusted HR, 0.90; 95% CI = 0.41–1.96; 6.4 vs. 7.3 per 1000 person-years) (Table 4).

## 4. Discussion

The results of this nationwide population-based cohort study showed that IBD patients treated with integrative therapy were significantly (0.56-fold) less likely to develop PD compared to IBD patients who received only monotherapy, after adjusting for sex, age, comorbidities, medication use, hospitalization (days), outpatient use (days), and number of hospitals visited in the population of South Korea. Moreover, the subgroup analysis showed that IBD patients with mild CCI treated with integrative therapy had a 0.39-fold lower risk of developing PD.

Previous nationwide population-based cohort studies have also investigated the increased risk of PD among IBD patients [8,9,10,11,12]. Lin et al. (2016) showed a 35% increase in the risk of PD in patients with IBD compared to that in patients without IBD in Taiwan, especially for CD [8]. In a US cohort study (2018), patients with IBD were 28% more likely to develop PD than were individuals without IBD and patients with IBD exposed to anti-tumor necrosis factor-alpha (TNF-α) agents had a 78% decrease in the risk of PD occurrence compared to the risk in unexposed patients [9]. However, neither study considered the number of hospitals visited, which might have resulted in surveillance bias effects on the risk of PD in patients with IBD. A Swedish cohort study (2018) reported a 30% increase in the risk of PD in patients with UC compared to that in controls, but these effects vanished after adjusting for the number of healthcare visits during the follow-up period to reduce potential surveillance bias [10]. A Danish cohort study (2018) also showed that patients with IBD had a 22% increase in the risk of PD as compared to the risk in non-IBD individuals [11]. The results have also led to debates over surveillance bias on the risk of PD in patients with IBD not considering the number of healthcare visits. Park et al. (2019) showed an increased risk of PD in patients with IBD in South Korea, which is an association that remained significant after adjusting for healthcare use. Furthermore, IBD patients receiving steroid or anti-TNF agents who may have had severe IBD showed a significantly reduced risk of PD compared to the risk in patients with mild disease [12].

The potential regulatory effect of cytokines on the growth and differentiation of the cells in the nervous system was reported by findings [22,23,24]. Based on these results, the association between neurons and immune system has continuously been investigated up to now. In the pathogenesis of IBD and PD, the inflammatory process that underlies both diseases shares some pro-inflammatory cytokines such as TNF-α and IL-1β. The central nigrostriatal dopaminergic degeneration followed by bowel inflammation is associated with increased TNF-α and IL-1β. Intestinal inflammation might be caused by alterations in the gut microbiota that regulate pathways that promote α-synuclein aggregation. α-Synuclein deposits present in the bowel wall may be disseminated through the vagus nerve and might also cause dopaminergic neurodegeneration in PD patients [25].

Currently, conventional medication therapy is used as the first option in the treatment of chronic and recurrent IBD for improving patients’ quality of life. According to a study investigating the medication use patterns in IBD patients based on data from a nationwide administrative claims-based database of the NHIS in South Korea, 5-aminosalicylic acid was most commonly used, followed by corticosteroids, immunomodulators, and anti-TNF-α agent [20]. However, long-term corticosteroid therapy for the treatment of IBD can lead to venous thromboembolism (VTE), infections, and fragility fractures [26]. The use of anti-TNF (infliximab and adalimumab) agents also significantly increases the risk of tuberculosis (TB) infection and malignancies [27,28].

Several studies investigated the efficacy and the mechanisms of KM treatment for the treatment of IBD. Acupuncture and moxibustion treatment showed better overall efficacy than 5-aminosalicylic acid in treating IBD [29]. According to a resting-state functional magnetic resonance imaging (fMRI) study in CD patients, electro-acupuncture and moxibustion mainly modulated the brain homeostatic afferent processing network and default mode network, respectively [30]. These results suggested that electro-acupuncture and moxibustion may help to improve clinical results in CD patients through different central integration patterns. Acupuncture and moxibustion also inhibited levels of IL-17 and retinoid-related orphan receptor gamma (RORγt) but induced forkhead box P3 (FOXP3) expression in the intestinal mucosa of patients with CD [31]. In addition, herb-partitioned moxibustion combined with acupuncture induces mucosa inflammation remission and repairs the tight junction barrier structure in intestinal epithelial mucosa, increasing the expression of zonula occludens-1 mRNA [32]. Another systematic review showed that complementary and alternative medicine (CAM) treatments integrated with conventional medicine might be safe and beneficial in IBD patients [33].

Acupuncture transmits signals into the vagus nerve and attenuates inflammatory responses, as measured by TNF-α productions in the serum and spleen. The vagus nerve acts as a bridge between the neural and internal organs for homeostatic regulation and immune systems [34]. Moxibustion also relieves visceral hypersensitivity and inhibits intestinal inflammation by regulating the relative abundance of the gut microbiota. Through these underlying mechanisms, acupuncture and moxibustion might be beneficial for IBD treatment and PD prevention [35].

In the present study, there were no significant differences between the integrative therapy and monotherapy groups among individuals with severe CCI. In the sensitivity analysis, Cox regression analysis showed more significant differences between the integrative therapy and monotherapy groups through KM ≥ 2, but the adjusted HR gradually increased for KM ≥ 3, ≥ 5, and ≥ 10 treatments. These findings might be explained by surveillance bias based on the following idea: “the more we look, the more we find” [36]. Unhealthy individuals are more concerned about their health so are more likely to use medical services, including KM treatment. Therefore, surveillance bias should be considered when evaluating outcome measures [37].

To our knowledge, this study is the first to compare the incidence of PD in IBD patients who received healthcare services other than conventional treatment. However, this study has several limitations. First, the NHIS-Senior cohort may have selection or ascertainment bias in health screening information due to issues regarding service eligibility. Furthermore, the results rely on the completeness and accuracy of data obtained from the nationwide administrative claims-based database of the NHIS. The disease codes listed in the cohort might not accurately represent the patients’ medical status. Second, information on potential confounding variables on personal health behaviors or lifestyle including physical activity, alcohol consumption, smoking, coffee drinking, socioeconomic status, occupation, and family medical history was limited because those data were obtained from self-reporting questionnaires in nationwide health screening. In addition, genetic factors including *CARD15/NOD2* and *LRRK2* were not evaluated due to data availability. The patterns of personal health behaviors and genetic factors have differed between the groups. Third, because the KM treatment patterns were mixed forms, such as acupuncture, moxibustion, cupping, psychotherapy and insured herbal preparations, the effect of each KM treatment could not be verified. Fourth, the effects of integrative treatment retention and adherence cannot be accurately quantified. Considering these limitations, the results of this study should be interpreted cautiously. Further investigations are needed to determine how KM treatment influences changes in the gut microenvironment and PD prevention. Long-term observational studies are also needed to determine the cumulative risk of PD in patients with IBD according to the KM treatment.

## 5. Conclusions

After adjusting for confounding variables, the results of this study showed that IBD patients in South Korea who received conventional and KM treatments had a significantly lower risk of PD compared to the risk in patients administered only conventional treatment, especially in those with mild CCI. Therefore, KM treatment may prevent PD in IBD patients. IBD patients should receive the KM treatment from the mild state of disease to prevent PD.

## Figures and Tables

**Table 1:** Baseline study population demographics.

	Monotherapy Group (*n* = 411)	Integrative Therapy Group (*n* = 1405)	*p*-Value
**Sex**			< 0.001
Male	242 (58.9%)	526 (37.4%)	
Female	169 (41.1%)	879 (62.6%)	
**Age**			0.061
60–64	167 (40.6%)	578 (41.1%)	
65–69	120 (29.2%)	419 (29.8%)	
70–74	63 (15.3%)	254 (18.1%)	
75–79	41 (36.7%)	121 (8.6%)	
≥80	20 (4.9%)	33 (2.4%)	
**Comorbidity**			
Alcohol-related diseases	20 (4.9%)	75 (5.3%)	0.706
Cardiovascular diseases	137 (33.3%)	580 (41.3%)	0.004
Chronic kidney diseases	44 (10.7%)	163 (11.6%)	0.615
Chronic obstructive pulmonary diseases	219 (53.3%)	881 (62.7%)	<0.001
Dementia	19 (4.6%)	74 (5.3%)	0.602
Depression	44 (10.7%)	230 (16.4%)	0.005
Diabetes mellitus	125 (30.4%)	467 (33.2%)	0.283
Hyperlipidemial	90 (21.9%)	364 (25.9%)	0.099
Hypertension	242 (58.9%)	853 (60.7%)	0.505
**Charlson Comorbidity Index**			<0.001
0	118 (28.7%)	246 (17.5%)	
1	128 (31.1%)	440 (31.3%)	
2	83 (20.2%)	378 (26.9%)	
≥3	82 (20.0%)	341 (24.3%)	
**Biomedicine Drug**			<0.001
Yes	299 (72.8%)	1132 (80.6%)	
No	112 (27.3%)	273 (19.4%)	
**Treatment period in biomedicine (days)**			0.207
1	204 (49.6%)	747 (53.2%)	
≥2	207 (50.4%)	658 (46.8%)	
**Hospitalization period in biomedicine (days)**			
0	360 (87.6%)	1281 (91.2%)	0.03
≥1	51 (12.4%)	124 (8.8%)	
**# of visiting biomedicine hospital**			0.158
1	362 (88.1%)	1271 (90.5%)	
≥2	49 (11.9%)	134 (9.5%)	

**Table 2:** Risk of PD in patients with IBD according to KM treatment.

	Monotherapy				Integrative Therapy					
	*n*	Events	Person-Year	Incidence ^a^	*n*	Events	Person-Year	Incidence	Crude HR (95% CI)	Adjusted ^b^ HR (95% CI)
**All**	411	25	2968	8.4	1405	55	10890	5.1	0.60 (0.37–0.96)	0.56 (0.34–0.92)
**Sex**										
Male	242	13	1718	7.6	526	19	3980	4.8	0.63 (0.31–1.28)	0.57 (0.28–1.17)
Female	169	12	1249	9.6	879	36	6910	5.2	0.54 (0.28–1.04)	0.58 (0.29–1.14)
**Age**										
<65	197	8	1607	5.0	664	20	5489	3.6	0.73 (0.32–1.66)	0.77 (0.33–1.82)
≥65	214	17	1361	12.5	741	35	5392	6.5	0.52 (0.29–0.92)	0.51 (0.28–0.94)
**Medication use**										
0	112	7	806	8.7	273	14	2084	6.7	0.77 (0.31–1.92)	0.79 (0.30–2.08)
1	299	18	2162	8.3	1132	41	8607	4.7	0.56 (0.32–0.97)	0.54 (0.30–0.96)

**^a^** Per 1000 person-years; **^b^** Model adjusted for sex, age, alcohol, cardiovascular, chronic kidney, chronic obstructive pulmonary disease, dementia, depression, diabetes mellitus, hyperlipdemial, hypertension, medication use, hospitalization medical use, outpatient medical use, and number of hospitals visited. HR: hazard ratio.

**Table 3:** Risk of PD in patients with IBD according to the number of KM treatments.

	Monotherapy				Integrative Therapy					
	*n*	Events	Person-Years	Incidence ^a^	*n*	Events	Person-Years	Incidence	Crude HR (95% CI)	Adjusted ^b^ HR (95% CI)
**Main analysis**										
# of KM ^c^ ≥ 1	411	25	2968	8.4	1405	55	10890	5.1	0.60 (0.37–0.96)	0.56 (0.34–0.92)
**Sensitivity analysis**										
# of KM ≥ 2	437	28	3170	8.8	1379	52	10688	4.9	0.55 (0.35–0.87)	0.51 (0.31–0.82)
# of KM ≥ 3	496	29	3613	8	1320	51	10245	5	0.62 (0.39–0.98)	0.57 (0.35–0.92)
# of KM ≥ 5	604	33	4412	7.5	1212	47	9447	5	0.66 (0.43–1.04)	0.62 (0.39–0.98)
# of KM ≥ 10	822	41	6105	6.7	994	39	7754	5	0.75 (0.48–1.16)	0.68 (0.43–1.07)

**^a^** Per 1000 person-years; **^b^** Model adjusted for sex, age, alcohol, cardiovascular, chronic kidney, chronic obstructive pulmonary disease, dementia, depression, diabetes mellitus, hyperlipdemial, hypertension, medication use, hospitalization medical use, outpatient medical use, and number of hospitals visited; **^c^** Number of Korean medicine treatments.

**Table 4:** Risk of PD in patients with IBD according to KM treatment in the CCI group.

	Monotherapy				Integrative Therapy				Crude HR (95% CI)	Model 1 ^a^	Model 2 ^b^
	*n*	Events	Person-Year	Incidence	*n*	Events	Person-Year	Incidence		Adjusted HR (95% CI)	
**CCI**											
Low	246	17	1872	9.1	686	20	5444	3.7	0.40 (0.21–0.77)	0.37 (0.19–0.71)	0.39 (0.20–0.77)
High	165	8	1096	7.3	719	35	5447	6.4	0.87 (0.41–1.89)	0.91 (0.42–1.99)	0.90 (0.41–1.96)

^a^ Model 1 adjusted for sex, and age; ^b^ Model 2 adjusted for sex, age, medication use, hospitalization medical use, outpatient medical use, and number of hospitals visited. CI: confidence interval.

## Supplementary Materials

The following are available online at https://www.mdpi.com/2077-0383/9/8/2422/s1, Table S1. Definitions of IBD drugs on the basis of main component codes and Table S2. Types of Korean medicine (KM) treatment.
