# Supplementary material for: The Impact of Korean Medicine Treatment on the Incidence of Parkinson’s Disease in Patients with Inflammatory Bowel Disease: A Nationwide Population-Based Cohort Study in South Korea
Source: J Clin Med. 2020 Jul 28;9(8):2422. doi: 10.3390/jcm9082422 (PMC7463832; doi:10.3390/jcm9082422)
Supplement: Supplementary file 1 [file jcm-09-02422-s001.pdf]

Supplementary Materials

Table S1. Definitions of IBD drugs on the basis of main component codes.

Family of drugs	Medications	Codes
**5-aminosalicylic acid**	mesalamine	190802ATR, 190803ATR, 190808ATR, 190811ATR, 190802CSP, 190803CSP, 190830CLQ, 190832CLQ
	sulfasalazine	232801ATE
	balsalazide	475001ACH
**Corticosteroid**	budesonide (micronized)	119501CMS
	hydrocortisone	170901ATB, 170906ATB, 171201BIJ
	prednisolone	217001ATB, 217034ASY, 217035ASY
	methylprednisolone	193302ATB, 193305ATB, 193601BIJ, 193603BIJ, 193604BIJ
	beclomethasone dipropionate	114511ATE
**Immunomodulator**	azathioprine	112401ATB, 112402ATB
	6-mercaptopurine	190601ATB
	methotrexate	192101ATB, 192132BIJ, 192134BIJ, 192136BIJ, 192144BIJ, 192142BIJ, 192143BIJ, 192139BIJ, 192141BIJ
	cyclosporine	139201ACS, 139204ACS, 194701ACS, 194702ACS, 194730ALQ, 139230BIJ
**Anti-TNF-α agent**	infliximab	383501BIJ
	adalimumab	488431BIJ, 488433BIJ, 488430BIJ

Table S2. Types of Korean medicine (KM) treatment.

KM	National Health Insurance EDI codes
**Acupuncture**	insertion of needle; needling; acupuncture (40011), insertion of needle; needling; acupuncture (more than 2 regions) (40012), insertion of needle; needling; acupuncture in orbit (40030), insertion of needle; needling; acupuncture in nasal cavity (40040), insertion of needle; needling; acupuncture in abdominal cavity (40050), intrarticular insertion of needle; needling; acupuncture (40060), intervertebral insertion of needle; needling; acupuncture (40070), piercing insertion (40080), electroacupuncture (40091, 40092), laser acupuncture (40100), scalp acupuncture/ ear acupuncture/ facial acupuncture/ nose acupuncture/ hand acupuncture therapy/ foot acupuncture therapy/ finger acupuncture therapy/ wrist ankle acupuncture/ tongue acupuncture (40120, 40131-40134)
**Moxibustion**	direct moxibustion (40304, 40305), indirect moxibustion (40306, 40307)
**Cupping**	retained cupping (40321), flash cupping (40322), slide cupping (40323), pricking and cupping; pricking with suction (40312), pricking and cupping; pricking with suction (more than 2 regions) (40313)
**Psychotherapy**	meridian skin and sinews hot pack therapy (40700), meridian cutaneous infrared radiating therapy (40701), meridian skin and sinews ice pack therapy (40702)
**Insured herbal preparations (single herb extracts)**	Pueraria Root extract powder (1001S1APD), Chrysanyhemum indicum extract power (1002S1APD), Glycyrrhiza extract powder (1003S1APD), Ostericum koreanum extract powder (1004S1APD), Ginger extract powder (1005S1APD), Cinnamomi ramulus extract powder (1006S1APD), Trichosanthes seed extract powder (1007S1APD), Agastachis Herba extract powder (1008S1APD), Lonicera Flower extract powder (1009S1APD), Platycodon root extract powder (1010S1APD), Korean Angelica extract powder (1011S1APD), Zizyphi fructus(jujube) extract powder (1012S1APD), Rhubarb extract powder (1013S1APD), Peach Kernel extract powder (1014S1APD), Aralia continentalis extract powder (1015S1APD), Ephedra sinica extract powder (1016S1APD), Viticis fructus extract powder (1017S1APD), Cynanchi Radix powder (1018S1APD), Liriopis Tuber extract powder (1019S1APD), Hordei Fructus extract powder (1020S1APD), Moutan Root Bark extract power (1021S1APD), Aucklandiae Radix extract powder (1022S1APD), Mentha herb extract powder (1023S1APD), Pinellia

	Tuber extract powder (1024S1APD), Saposhnikovia Root extract powder (1025S1APD), Ginseng extract powder (1026S1APD), Angelica dahurica Root extract powder (1027S1APD), Atractylodes Rhizome White extract powder (1028S1APD), Hoelen extract powder (1029S1APD), Zedoaria rhizome extract powder (1030S1APD), Amomum Fruit extract powder (1031S1APD), Hawthorn Fruit extract powder (1032S1APD), Scirpi Rhizoma extract powder (1033S1APD), Ginger extract powder (1034S1APD), Rehmannia Root extract powder (1035S1APD), Gypsum powder (1036S1APD), Asiasari Radix extract powder (1037S1APD), Perilla Herb extract powder (1038S1APD), Steamed rehmannia root extract powder (1039S1APD), Cimicifuga Rhizome extract powder (1040S1APD), Bulpleurum root extract powder (1041S1APD), Massa Medicata Fermentata extract powder (1042S1APD), Forsythia Fruit extract powder (1043S1APD), Schizandra Fruit extract powder (1044S1APD), Cinnamomi Cortex Spissus extract powder (1045S1APD), Artemisiae herba extract powder (1046S1APD), Paeony Root extract powder (1047S1APD), Anthriscus sylvestris exract powder (1048S1APD), Aurantii Fructus Immaturus exract powder (1049S1APD), Anemarrhena Rhizome extract powder (1050S1APD), Poncirus Fruit extract powder (1051S1APD),<br>Citrus unshiu Peel extract powder (1052S1APD), Atractylodes Rhizome extract powder (1053S1APD), Cnidium Rhizome extract powder (1054S1APD), Gastrodia Rhizome extract powder (1055S1APD), Asparagus Tuber extract powder (1056S1APD), Aurantii immaturi Pericarpium extract powder (1057S1APD), Gardenia Fruit extract powder (1058S1APD), Alismatis Rhizoma extract powder (1059S1APD), Apricot Kernel extract powder (1060S1APD), Cyperus Rhizome extract powder (1061S1APD), Schizonepeta Spike extract powder (1062S1APD), Scutellaria Root extract powder (1063S1APD), Astragalus Root extract powder (1064S1APD), Coptis Rhizome extract powder (1065S1APD), Phellodendron bark extract powder (1066S1APD), Magnolia Bark extract powder (1067S1APD)
**Insured herbal preparations (complexed herb extracts)**	Gamisoyosan (2001M1APD), Galgeuntang (2002M1APD), Galgeunhaegitang (2003M1APD), Goomiganghwaltang (2004M1APD), Goongsosan (2005M1APD), Gunghatang (2006M1APD), Naesosan (2007M1APD), Dangguiyungyoeum (2008M1APD), Dangguiyoughwangtang (2009M1APD), Daesihotang (2010M1APD), Daechungryongtang (2011M1APD), Daehwajoongum (2012M1APD), Daehwangmogdanpitang (2013M1APD), Doinsungkitang (2014M1APD), Banhabaegchoolcheonmatang (2015M1APD), Banhasasimtang (2016M1APD), Banhahoobagtang (2017M1APD), Baekchultang (2018M1APD), Bojungikgitang (2019M1APD), Boheotang (2020M1APD), Bokryengbosimtang (2021M1APD), Boolhwankumjeongkisan (2022M1APD), Samsoeum (2023M1APD), Samchoolkeonbitang (2024M1APD), Samhojagyagtang (2025M1APD), Samhwangsasimtang (2026M1APD), Sengmeksan (2027M1APD), Sosihotang (2028M1APD), Socheongryongtang (2029M1APD), Seungyangbowitang (2030M1APD), Sikyengbanhatang (2031M1APD), Sihogyejitang (2032M1APD), Sihosogantang (2033M1APD), Sihocheongkantang (2034M1APD), Antaeum (2035M1APD), Yunkyopaedocksan (2036M1APD), Orimsan (2037M1APD), Ojeoksan (2038M1APD), Ijoongtang (2039M1APD), Ijintang (2040M1APD), Inwisungyangtang (2041M1APD), Insampaedoksan (2042M1APD), Injinhotang (2043M1APD), Jaumkanghwatang (2044M1APD), Jowiseunggitang (2045M1APD), Chungsanggyuntongtang (2046M1APD), Cheogseoikgitang (2047M1APD), Cheongwisan (2048M1APD), Palmooltang (2049M1APD), Pyengwisan (2050M1APD), Haengsotang (2051M1APD), Hyangsapeungeuysan (2052M1APD), Hwangkumjagyagtang (2053M1APD), Hwangryenhaedogtang (2054M1APD), Hyunggaeyenkyotang (2055M1APD), Hwichoonyangkyegsan (2056M1APD)
